# A Sensation in Medical Circles

**Published:** 1888-10

**Authors:** 


A SENSATION IN MEDICAL CIRCLES has been created in Memphis by the detection of an ex-Professor in the Memphis Hospital Medical College, Dr. J. P. McGee, formerly of Trenton, Tenn., in an attempt to manufacture a counterfeit of the diploma used by the Memphis college.

Dr. McGee came to Memphis in 1883 by invitation of the Faculty, he being a prominent member of the State Medical Association, and we believe, formerly, its president; and accepted the chair of Materia Medica and Practice. This position he held till '85, when he resigned, under charges preferred by Prof. Rogers, for unprofessional conduct. The facts, as given in the Memphis Avalanche, are briefly as follows: McGee went to a stencil cutter with a borrowed (?) diploma of a Dr. Voorhies, and, showing it to the artist, requested him to counterfeit the seal. The workman refused, but Dr. Rogers getting wind of the affair in some way, had a detective to shadow McGee. The detective induced the artist to go after McGee, and tell him he had consented to make the seal. McGee accordingly left the bogus diploma, to which the seal was to be affixed,—in the shop, and the detective got it and showed it to Dr. Rogers. It was arranged for the detective to pounce on the doctor in the act of affixing the seal, but he smelt a mouse and sent, two days in advance, for the manufactured diploma—and thus he escaped conviction. The diploma was, to all appearances, made out on one of the parchment sheets used by the college, and which McGee must have secured during his connection with the college. The signatures were exact imitations of those of the Faculty, and pronounced a forgery by the Dean. The bogus diploma was made out in beautiful scroll work, in the name of one "Dr. B. C. Kelsey," now of Denver, a once notorious advertising doctor, at Chelsea, Ark. McGee denies that he was to receive anything for his services—but—where was the incentive to attempt such a crime? At one time no man stood fairer in medical circles in Tennessee than Dr. McGee. Now, he has fallen too low to be kicked. Alas!

